# Thermoresponsive Magnetic Nano-Biosensors for Rapid Measurements of Inorganic Arsenic and Cadmium

**DOI:** 10.3390/s121014041

**Published:** 2012-10-18

**Authors:** Mohammad Shohel Rana Siddiki, Shun Shimoaoki, Shunsaku Ueda, Isamu Maeda

**Affiliations:** 1 United Graduate School of Agricultural Science, Tokyo University of Agriculture and Technology, 3-5-8 Saiwaicho, Fuchu 183-8509, Japan; E-Mails: msrsiddiki@yahoo.com (M.S.R.S.); uedashun@cc.utsunomiya-u.ac.jp (S.U.); 2 Faculty of Agriculture, Utsunomiya University, 350 Minemachi, Utsunomiya 321-8505, Japan; E-Mail: metal_gear1964@yahoo.co.jp

**Keywords:** magnetic nanoparticles, heavy metal, biosensor, ArsR, CadC, GFP

## Abstract

Green fluorescent protein-tagged sensor proteins, ArsR-GFP and CadC-GFP, have been produced as biosensors for simple and low-cost quantification of As(III) or Cd(II). In this study, the sensor protein-promoter DNA complexes were reconstructed on the surfaces of magnetic particles of different sizes. After the surface modification all the particles could be attracted by magnets, and released different amounts of GFP-tagged protein, according to the metal concentrations within 5 min, which caused significant increases in fluorescence. A detection limit of 1 μg/L for As(III) and Cd(II) in purified water was obtained only with the nanoparticles exhibiting enough magnetization after heat treatment for 1 min. Therefore, thermoresponsive magnetic nano-biosensors offer great advantages of rapidity and sensitivity for the measurement of the toxic metals in drinking water.

## 1. Introduction

Magnetic separation and nanotechnology have attracted much attention, and could be applied potentially to various fields. Their superior features meet technological and industrial requirements. Magnetic nanoparticles (MNPs) have been applied to biotechnological fields because the magnetism phenomenon and nano-size diameter of particles enable the efficient capture of biological molecules and easy separation of captured molecules from liquid phases [1–3]. Magnetic particles can be bound not only to biologically active molecules such as peptides, receptors, ligands, and antibodies [4], but also to oligonucleotides via streptavidin-biotin conjugation [5]. MNPs usually have very short sizes, and, therefore, in several cases, it is very difficult to collect the particles magnetically. The thermoresponsive MNPs start to flocculate by heating, and this makes the particles separate from the solution within a short time using a magnet [6].

Solid phase biosensors have been developed by modifying the surface of a microplate well with a complex of green fluorescent protein (GFP)-tagged sensor protein, ArsR-GFP or CadC-GFP, and their *cis* element [7]. By simply adding a drinking water sample to the well, GFP-tagged sensor protein became dissociated from *cis* element, and this caused significant differences in fluorescence intensity within 15 min for Cd(II) or 30 min for As(III), depending on their concentrations. The recombinant transcriptional regulator proteins fused to GFP could associate to oligonucleotides through molecular recognition for specific nucleotide sequences [8]. Therefore, thermoresponsive MNPs may be substituted for microplate wells as a scaffold for the solid phase biosensors.

**Figure 1.**
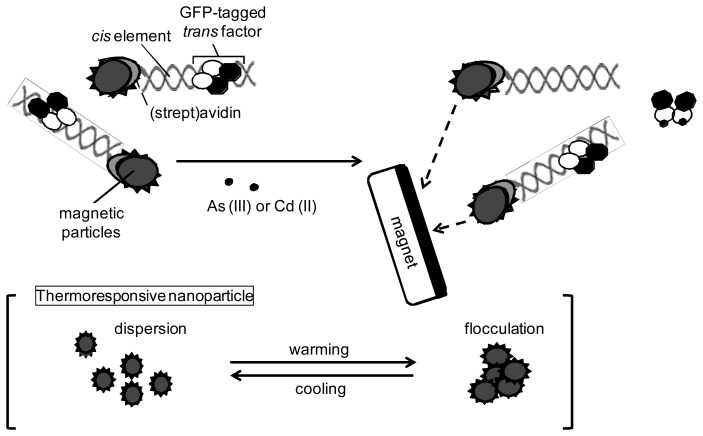
Schematic presentation of principle involved in the solid phase biosensors constructed on magnetic particles. In the case of thermoresponsive nanoparticles, flocculation was needed for magnetic separation.

In this study, complexes composed of sensor protein−promoter DNA were reconstructed on the surface of different types of magnetic particles. Once toxic metal ions bound to the sensor protein, its capability of binding to the promoter DNA was lost. Therefore, magnetic separation of the free sensor protein at metal-binding conformation from the residual protein at DNA-binding conformation could be considered (Figure 1). It was examined whether use of magnetic particles as a scaffold of the complexes composed of sensor protein and promoter DNA is effective for reduction of assay time and, if so, which particles are suitable for rapid and sensitive determination.

## 2. Experimental Section

### 2.1. Preparation of Magnetic Biosensors

Cell lysates containing GFP-tagged *trans* factor were prepared using *E. coli* BL21 (DE3) pLysS (Novagen–Merck, Darmstadt, Germany) harboring pETarsR–gfp or pETcadC–gfp [8], as described previously. The double-stranded *ars* operator, *O*_ars_−30-down [7], and the double-stranded *cad* promoter−operator, *P*_**cad**_−*O*_**cad**_ [8], were prepared as shown previously.

Two types of magnetic particles, Streptavidin Mag Sepharose (GE Healthcare, Tokyo, Japan) and Dynabeads M-280 streptavidin (Life Technologies, Tokyo, Japan), and one type of thermoresponsive nanoparticles, Therma-max LA avidin (JNC, Tokyo, Japan), were used in this study. Each suspension was mixed thoroughly by vortexing, and 50 μL of the suspension was dispensed into 1.7 mL polypropylene conical tubes. The storage solution was removed after placing the tubes on a magnetic stand and making particles accumulate on the side wall of tubes. In case of the thermoresponsive nanoparticles, the storage solution was removed after incubating the tubes at 42 °C for 1 min, because the aggregation of the particles is more rapid above the lower critical solution temperature (21 °C) of the coated polymer [6]. Then, the particles were resuspended in 250 μL of 25 mM Tris-HCl buffer pH 7.4 for equilibration, which was removed in the same way as shown above.

The equilibrated particles were resuspended in 20 picomol per 150 μL of double-stranded DNA fragment in 25 mM Tris-HCl buffer pH 7.4 and incubated for immobilization of it onto the particles at room temperature for 30 min. After immobilization, excess unbound DNA was removed and the DNA-immobilized particles were washed off three times by 25 mM Tris-HCl buffer pH 7.4. The washing buffer was mixed with the DNA-immobilized particles and removed while holding the particles in the tube by magnet. One hundred and fifty microliters each of ArsR-GFP or CadC-GFP mixture, prepared as shown previously [7], was poured into 1.7 mL polypropylene conical tubes, in which the DNA-immobilized magnetic particles were included, and incubated for 30 min. Free unbound protein was removed and the protein-bound particles were washed off once with 150 μL of 25 mM Tris-HCl buffer pH 7.4 while holding the particles in the tube by magnet. In case of the thermoresponsive nanoparticles, removal of excess unbound DNA or protein and washing were performed after incubating the tubes at 42 °C for 1 min. Magnetic biosensors were ready-to-use after removal of unbound protein and one-time use for toxic-metal assay.

### 2.2. As(III) or Cd(II) Assay Protocol

Sample mixtures with and without As(III) or Cd(II) were prepared using 98% NaAsO_2_ and CdCl_2_·2.5H_2_O (both from Sigma-Aldrich, Tokyo, Japan) in ultrapure water (Simplicity UV, Millipore, Tokyo, Japan). For the As(III) assay, 93.5 volumes of sample was mixed with 5 volumes of 1 M potassium phosphate buffer (KPB) pH 6.7, 0.5 volumes of 10 mg/mL salmon sperm DNA, and 1 volume of 4 M NaCl (final concentrations; 50 mM KPB, 50 μg/mL salmon sperm DNA, 40 mM NaCl). For the Cd(II) assay, sample mixtures were mixed with a similar composition except for that 1 M Tris-HCl pH 7.9 was used instead of 1 M KPB. One hundred and fifty-five microliters of sample mixture was added to each tube, in which the protein-bound particles were included, and incubated for the time indicated in the relevant figure legends, to release GFP-tagged *trans* factor binding to metal from immobilized *cis* element (Figure 1). Particles were collected with a magnetic stand to recover the supernatant. The thermoresponsive nanoparticles acquired magnetism after warming at 42 °C for the time indicated in the relevant figure legends. Then, 150 μL of the supernatant containing dissociated protein binding with metals was transferred to black plate wells and fluorescence intensity was measured with a microplate fluororeader at excitation/emission wavelengths of 490/530 nm (MTP-601, Hitachi high technologies, Tokyo, Japan). One hundred and forty-five microliters of the supernatants were transferred to glass vials to measure with a handheld, battery-powered portable fluorometer (GFP-pen GFP 100, Photon systems instruments, Brno, Czech Republic). The toxic metals were measured individually because the sensor proteins, ArsR-GFP and CadC-GFP, had different selectivity to metals and could not be distinguished individually. The selectivity of the sensor proteins to other metals and interference to their responses by minerals in water have been precisely reported elsewhere [8].

### 2.3. Evaluation of Magnetic Driving

One hundred microliters of Mag Sepharose and Dynabeads were taken to prepare the magnetic biosensors, on which *P*_cad_−*O*_cad_ was immobilized or the complex of CadC-GFP/*P*_cad_−*O*_cad_ was constructed. Finally, suspensions of the microparticles with or without the surface-modifications were filled up to 800 μL with 25 mM Tris-HCl pH 7.4. Each suspension was transferred to a cuvette and optical density at 600 nm (OD_600_) was measured with a spectrophotometer (U-2910, Hitachi High Technologies, Tokyo, Japan). A magnet was placed at 13 mm from a cuvette holder in an experiment using Mag Sepharose while it was attached directly to the holder in an experiment using Dynabeads because Mag Sepharose particles were so quickly driven by an attached magnet that the data could not be obtained without adjusting its position. Fluorescence intensity of the three types of particles was measured using a black microplate and fluororeader (MTP-601, Hitachi High Technologies, Tokyo, Japan).

### 2.4. Evaluation of Thermoresponsive Flocculation Properties

One hundred microliters of Therma-max was taken to prepare the magnetic nano-biosensor, as described in the experiment for evaluation of magnetic driving. Each suspension was transferred to a cuvette and placed in a spectrophotometer equipped with an air-cooled Peltier thermo-panel cell holder (V-630 and EHC-716, both from JASCO, Tokyo, Japan) to control temperature of suspension in the cuvette. The incubation temperature was increased from 21 °C to 37 °C to evaluate thermoresponsive flocculation of nanoparticles with absorbance at 420 nm (A_420_) [6]. Fluorescence intensity of the three types of particles was measured as described in the experiment for evaluation of magnetic driving.

## 3. Results and Discussion

### 3.1. Responses of the Solid Phase Biosensor to Toxic Metals

**Figure 2.**
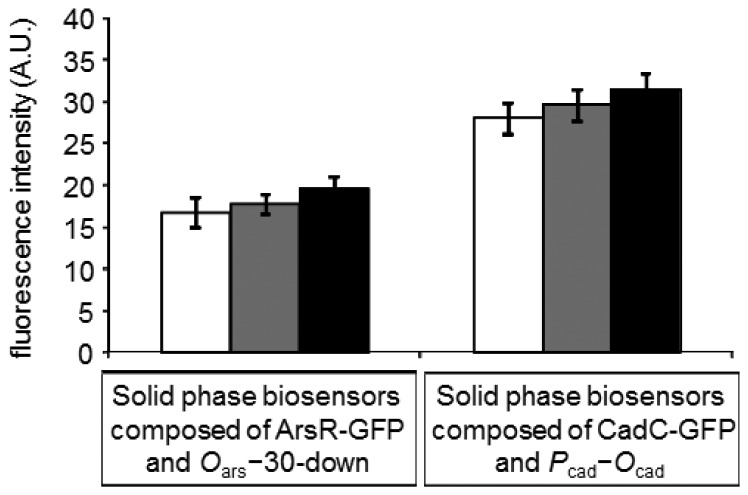
Fluorescence changes after 5-min incubation with sample on the solid phase biosensors constructed on the surface of microplate wells in response to As(III) (left bars) and Cd(II) (right bars). White bars shows intensities obtained without addition of the toxic metals, whereas gray and black bars show those obtained at concentrations of 10 μg/L and 100 μg/L, respectively. Fluorescence was measured by fluorometer.

When the solid phase biosensors, in which ArsR-GFP/*O*_ars_−30-down or CadC-GFP/*P*_cad_–*O*_cad_ was constructed on the surface of microplate wells, were tested under assay conditions of 5-min incubation, no significant responses to As(III) and Cd(II) were observed (Figure 2). The result indicated the difficulty to complete the assay within 5 min if the solid phase biosensors would be constructed on the surface of microplate wells. Therefore, the three types of commercially available magnetic particles were separately examined as a scaffold of the solid phase biosensors.

### 3.2. Properties of the Surface-Modified Magnetic Particles

**Figure 3.**
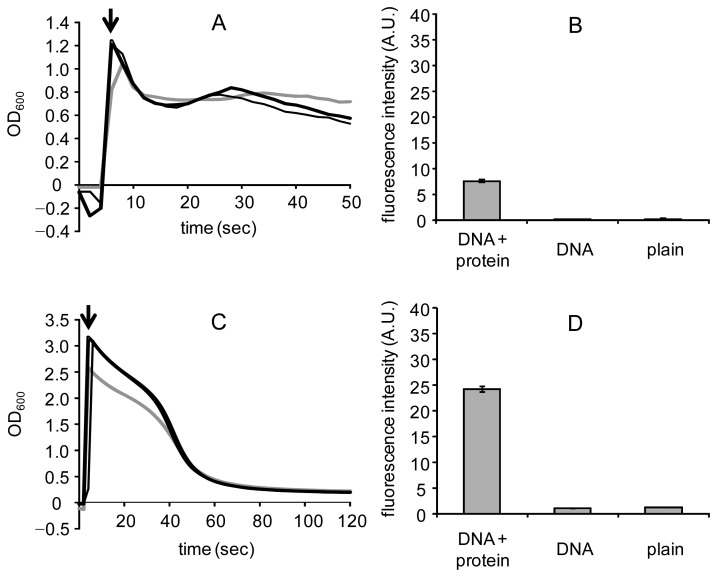
Effects of the surface modification of Mag Sepharose (**A**) and Dynabeads (**C**) plain particles (grey line) with DNA (black line) or DNA and protein (bold black line) on magnetic driving. Arrow shows the start of measurement. Fluorescence intensities of the Mag Sepharose (**B**) and Dynabeads (**D**) particles modified with DNA and protein.

**Figure 4.**
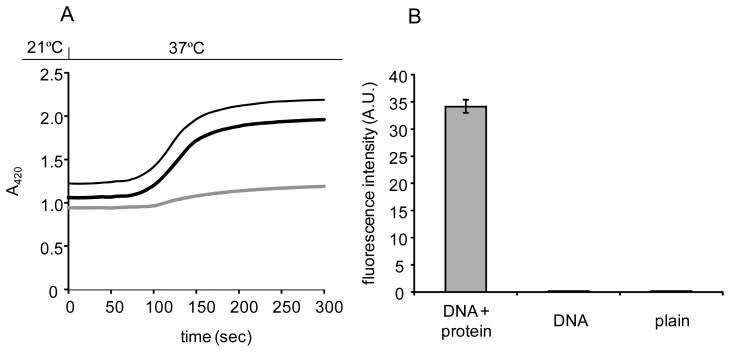
Effects of the surface modification of Therma-max plain particles (grey line) with DNA (black line) or DNA and protein (bold black line) on thermoresponsive flocculation (**A**). Fluorescence intensity of the particles modified with DNA and protein (**B**).

Mag Sepharose particles were quickly attracted to the magnet, and it resulted in decreases in OD_600_ within 15 s, regardless of the presence and absence of surface modification (Figure 3(A)). Dynabeads particles showed moderate decreases in OD_600_, which suggested almost all the particles were attracted to magnet within 60 s (Figure 3(C)). The fluorescence intensities were significantly higher in DNA/protein-modified particles than in DNA-immobilized or plain particles (Figure 3(B,D)). These data indicated that the magnetic biosensors were successfully constructed and the modification of DNA or DNA/protein did not affect magnetic driving of the particles. When Therma-max particles were modified with either *P*_cad_−*O*_cad_ or CadC-GFP/*P*_cad_−*O*_cad_, their marked thermoresponsive flocculation took place after 75 s and completed within 300 s (Figure 4(A)). A larger heat capacity of the suspension in a cuvette might cause a delay of flocculation, compared to the cases of DNA-immobilization and DNA/protein modification where recovery of the particles completed after 1 min of heat treatment. The highest fluorescence intensity in DNA/protein-modified particles was obtained using Therma-max (Figure 4(B)), indicating efficient construction of the DNA−protein complex on the thermoresponsive magnetic nano-biosensors. Therefore, the surface modification of MNPs with promoter−operator DNA and sensor protein and thermoresponsive flocculation, key factors to reduce assay time, were confirmed.

### 3.3. Detection of As(III) with the Magnetic Biosensors

**Figure 5.**
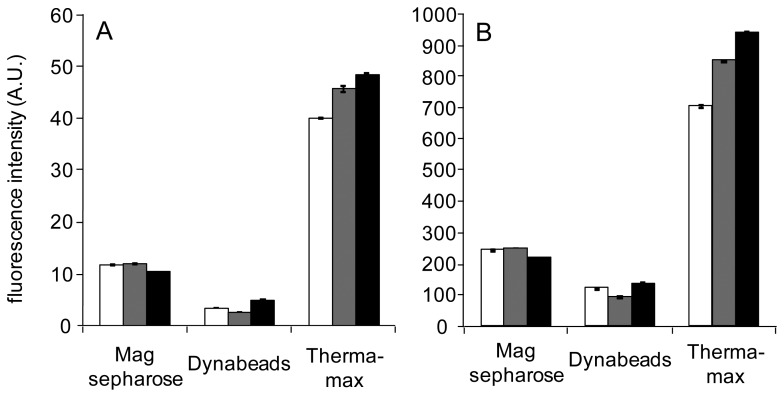
Fluorescence responses of the magnetic biosensors constructed on different types of particles to As(III) after a 5-min incubation. Fluorescence of ArsR-GFP released from *O*_ars_−30-down was measured by fluororeader (**A**) and fluorometer (**B**). White bars show intensities obtained without addition of As(III), whereas gray and black bars show those obtained at concentrations of 10 μg/L and 100 μg/L As(III), respectively. Incubation for 1 min at 42 °C was included in case of Therma-max.

**Figure 6.**
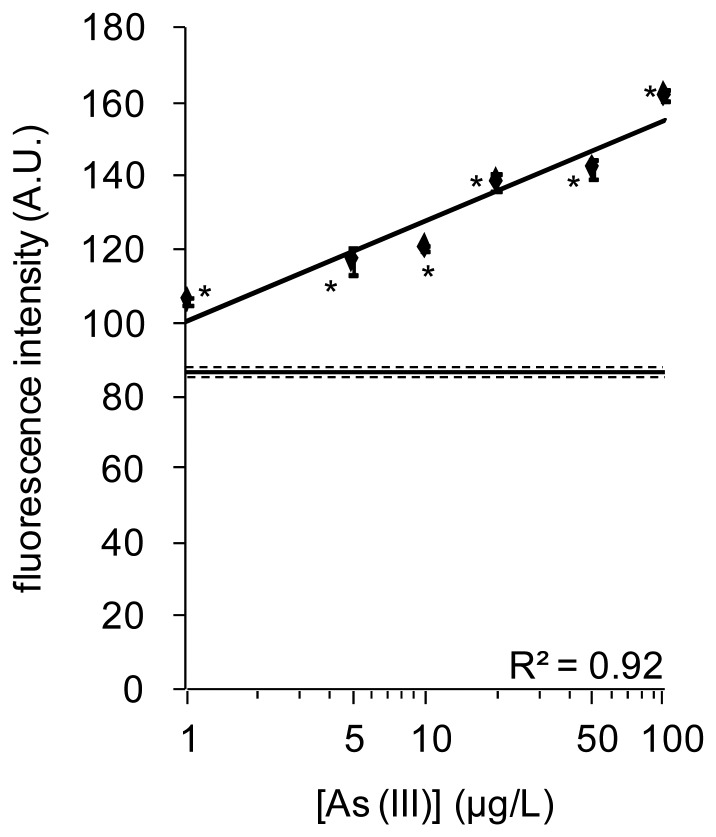
A dose-response of ArsR-GFP on the thermoresponsive magnetic nano-biosensor to As(III) concentration after a 5-min incubation including a 1-min treatment at 42 °C. The solid line and two broken lines show mean ± SD. Statistical significance (*P* < 0.001) against data obtained in water without addition of As(III) was shown with asterisk.

Magnetic biosensors to detect As(III) were constructed by modification of different types of magnetic particles with a complex of ArsR-GFP and *O*_ars_−30-down. When fluorescence intensity was measured with a fluororeader and fluorometer, significant responses to 10 and 100 μg/L As(III) were observed using Therma-max (Figure 5). Using Dynabeads, however, a significant response was obtained only at 100 μg/L, and no significant increases were observed using Mag Sepharose. Dissociation of ArsR-GFP from DNA-immobilized Therma-max was higher in 100 μg/L than in 10 μg/L (Figure 5). This indicates that quantification of As(III) with a complex of ArsR-GFP and *O*_ars_−30-down completes after a 4-min incubation with the sample mixture and a 1-min incubation at 42 °C when Therma-max LA avidin was used as a scaffold of it. Therefore, the dose-response of fluorescence intensity to As(III) concentration was investigated with Therma-max and fluorometer (Figure 6). A dose-dependent increase of fluorescence was observed at concentrations of 20 μg/L and less, and a detection limit of 1 μg/L was achieved using the thermoresponsive magnetic nano-biosensor comprising ArsR-GFP and *O*_ars_−30-down within 5 min. These features were superior to those of the solid phase biosensor, in which the sensing complex was constructed on the surface of microplate wells. Moreover, the results also indicated that this nano-biosensor had linearity between 1 and 100 μg/L, and reproducibility between different ArsR-GFP batches in detection of As(III). Therefore, the thermoresponsive MNPs could offer reduction of the detection time and detection limit in the As(III) assay.

### 3.4. Detection of Cd(II) with the Magnetic Biosensors

**Figure 7.**
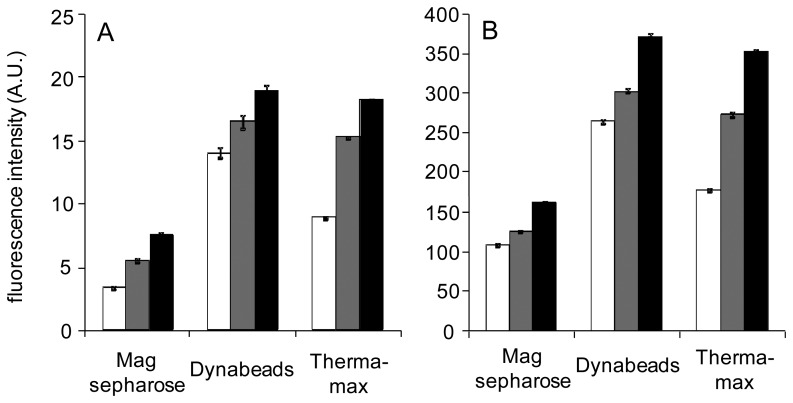
Fluorescence responses of the magnetic biosensors constructed on different types of particles to Cd(II) after a 1-min incubation. Fluorescence of CadC-GFP released from *P*_cad_−*O*_cad_ was measured by fluororeader (**A**) and fluorometer (**B**). White bars shows intensities obtained without addition of Cd(II), whereas gray and black bars show those obtained at concentrations of 10 μg/L and 100 μg/L Cd(II), respectively. Incubation for 2 min at 42 °C was added in case of Therma-max.

**Figure 8.**
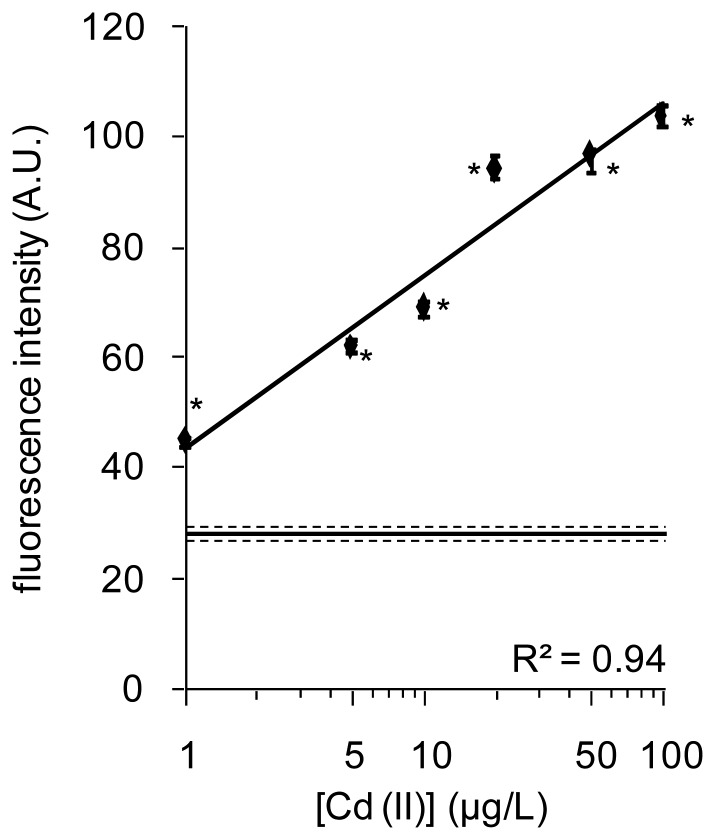
A dose-response of CadC-GFP on the thermoresponsive magnetic nano-biosensor to Cd(II) concentration after 2-min incubation including 1-min treatment at 42 °C. The solid line and two broken lines show mean ± SD. Statistical significance (*P* < 0.001) against data obtained in water without addition of Cd(II) was shown with asterisk.

Sample mixtures containing different concentrations of Cd(II) were poured into the tubes, in which one of the magnetic biosensors comprising a complex of CadC-GFP/*P*_cad_–*O*_cad_ was included. The magnetic biosensors were examined to determine whether dissociation of CadC-GFP from *P*_**cad**_−*O*_**cad**_ is enhanced by Cd(II) within 1 min of incubation (Figure 7). The fluorescence intensities, regardless of the measurement devices and magnetic particles, increased with increasing Cd(II) concentration. Fluorescence increase at 10 μg/L was more marked with Therma-max than with the other two brands. However, Therma-max required heat treatment for 1 to 2 min before recovery of the particles. As removal of the nanoparticles and sharp fluorescence responses to 10 μg/L and 100 μg/L Cd(II) were observed, dose-responses of fluorescence to Cd(II) concentration were examined with Therma-max and fluorometer. After a 1-min incubation at room temperature and 1-min incubation at 42 °C, a detection limit of 1 μg/L Cd(II) was achieved in ultrapure water and the dose-dependence was found at concentrations of 20 μg/L and less (Figure 8). Moreover, the results also indicated that this nano-biosensor had linearity between 1 and 100 μg/L and reproducibility between different CadC-GFP batches in the detection of Cd(II). Therefore, use of the thermoresponsive MNPs was advantageous to reduce the incubation time of the solid phase biosensor with water samples in monitoring of Cd(II).

### 3.5. Comparison of the Detection Limits and Recovering Efficiencies among the Particles with Different Size

Particle sizes were 90 nm for Therma-max, 2.8 μm for Dynabeads, and 37 to 100 μm for Mag Sepharose. The detection limits and efficiencies of magnetic driving seemed to be dependent on their sizes. A series of Mag Sepharose particles were quickly attracted to the magnet, whereas at a concentration of 10 μg/L, ArsR-GFP associated to the particles could not produce a significant difference in fluorescence intensity in response to As(III) or responded weakly to Cd(II). On the other hand, the thermoresponsive nano-biosensors exhibited a detection limit as low as 1 μg/L both for As(III) and Cd(II), whereas a series of Therma-max particles required an extra heat treatment to enable magnetic recovery by producing flocculation, as supposed from the report that the saturation magnetization decreases with decreasing size also in proportion to the specific surface area of the particles [9]. The lower detection limit of thermoresponsive nano-biosensors within short incubation times is explained by their high surface area to volume ratio, which might provide them with enough surface-modifying capacity and rapid molecule-interacting kinetics [10]. When the rapid assays for low concentrations of toxic metals are more intensively focused, construction of the thermoresponsive nano-biosensors will be more reasonable than application of the magnetic microparticles to biosensor construction.

### 3.6. Advantages and Disadvantages of the Method

Various methods have been commonly used for determination and quantification of metals [11,12]. Although most of them are not applicable for on-site measurement at low concentrations, voltammetric techniques such as square wave and stripping voltammetry are rapid procedures, and possess very low detection limits [0.02 μg/L As(III) and 0.04 μg/L Cd(II)] [12]. On the other hand, a detection limit of thermoresponsive magnetic nano-biosensors [1 μg/L for As(III) or Cd(II)] was higher than those of volammetry. However, the detection limit of nano-biosensors is still below the guideline value of arsenic in drinking water set by the World Health Organization and, therefore, the method is of practical use. Advantages of the fluorescence method are lower initial cost and portability of all the components, including a handheld battery-powered fluorometer, compared with those of electrochemical devices, and this should broaden the range of applications. Under consideration of detecting the toxic metals in turbid samples such as foods with the fluorescent method, however, pre-treatment procedures to extract metals from the samples into water would be required, as demonstrated in the detection of Pb(II) in soil [8].

## 4. Conclusions

Within 5 min, GFP-tagged sensor proteins, ArsR-GFP and CadC-GFP, dissociated from promoter−operator DNA on magnetic particles could successively cause significant increases in fluorescence intensity with increasing As(III) and Cd(II) concentrations, respectively. Among magnetic particles tested, the thermoresponsive nanoparticles had an advantage in detection for 10 μg/L As(III) or Cd(II), at which the other magnetic microparticle-based biosensors were not responsive. The dose-responses of thermoresponsive magnetic nano-biosensors (T-MNBs) showed the detection limit of 1 μg/L within 2 min for Cd(II) and within 5 min for As(III) using a battery-powered portable fluorometer. Disadvantages of T-MNB, compared to the other magnetic micro-biosensors tested, were that an additional incubation stage was required for recovery and, even after flocculation took place, only weak attractive forces to the magnet were generated. Even though these disadvantages should be considered, the merits of T-MNB in terms of detection time and detection limit should have great impact on the introduction of magnetic particles as a scaffold in solid phase biosensors.
